# Sphingomyelin synthases 1 and 2 exhibit phosphatidylcholine phospholipase C activity

**DOI:** 10.1016/j.jbc.2021.101398

**Published:** 2021-11-10

**Authors:** Yeun-po Chiang, Zhiqiang Li, Yang Chen, Yu Cao, Xian-Cheng Jiang

**Affiliations:** 1 Department of Cell Biology, SUNY Downstate Health Sciences University, Brooklyn, New York, USA; 2 Molecular and Cellular Cardiology Program, VA New York Harbor Healthcare System, Brooklyn, New York, USA; 3 Institute of Precision Medicine, Ninth People's Hospital, School of Medicine, Shanghai Jiao Tong University, Shanghai, China

**Keywords:** phosphatidylcholine phospholipase C (PC-PLC), sphingomyelin synthase 1 and 2, phosphatidylcholine, diacylglycerol, sphingomyelin, AdV, adenovirus, CPE, ceramide phosphorylethanolamine, D609, tricyclodecan-9-yl-potassium xanthate, DAG, diglyceride, dKO, double KO, NBD, nitrobenz-diazol, PC, phosphatidylcholine, P-choline, phosphorylcholine, PE, phosphatidylethanolamine, P-ethanolamine, phosphorylethanolamine, PLC, phospholipase C, rSMSs, recombinant SMSs, SMS1 and SMS2, sphingomyelin synthase 1 and 2, SMSr, sphingomyelin synthase–related protein, tKO, triple KO

## Abstract

Many studies have confirmed the enzymatic activity of a mammalian phosphatidylcholine (PC) phospholipase C (PLC) (PC-PLC), which produces diacylglycerol (DAG) and phosphocholine through the hydrolysis of PC in the absence of ceramide. However, the protein(s) responsible for this activity have never yet been identified. Based on the fact that tricyclodecan-9-yl-potassium xanthate can inhibit both PC-PLC and sphingomyelin synthase (SMS) activities, and SMS1 and SMS2 have a conserved catalytic domain that could mediate a nucleophilic attack on the phosphodiester bond of PC, we hypothesized that both SMS1 and SMS2 might have PC-PLC activity. In the present study, we found that purified recombinant SMS1 and SMS2 but not SMS-related protein have PC-PLC activity. Moreover, we prepared liver-specific *Sms1*/global *Sms2* double-KO mice. We found that liver PC-PLC activity was significantly reduced and steady-state levels of PC and DAG in the liver were regulated by the deficiency, in comparison with control mice. Using adenovirus, we expressed *Sms*1 and *Sms*2 genes in the liver of the double-KO mice, respectively, and found that expressed SMS1 and SMS2 can hydrolyze PC to produce DAG and phosphocholine. Thus, SMS1 and SMS2 exhibit PC-PLC activity *in vitro* and *in vivo*.

Phosphatidylcholine (PC) is an essential phospholipid for cell formation, growth, and death (1). The steady-state levels of PC should be controlled by its biosynthesis (Kennedy pathway) and catabolic pathways, including PC-phospholipase C (PC-PLC) (2, 3). PLCs are a group of enzymes that produce a diacylglycerol (DAG) and a phosphorylated molecule, such as phosphorylcholine (P-choline) and phosphorylethanolamine (P-ethanolamine) (4). It is conceivable that PC-PLC activity is important in maintaining steady-state levels of PC, thus influencing cell membrane integrity and function.

Although bacterial PC-PLC was cloned previously (5), the gene, which is responsible for mammalian PC-PLC, is still unknown so far. Therefore, PC-PLC studies in both mammalian cells and *in vivo* have to rely on PC-PLC inhibitors, such as tricyclodecan-9-yl-potassium xanthate (D609) (6).

Mammalian sphingomyelin synthase (SMS) family contains three members (7, 8). SMS1 and SMS2 can transfer P-choline from PC onto ceramide to form SM. SMS-related protein (SMSr) has no SMS activity; however, *in vitro*, it can transfer P-ethanolamine from phosphatidylethanolamine (PE) onto ceramide to form ceramide P-ethanolamine (CPE), an SM-like molecule (9). While SMS activity uses ceramide as the acceptor for the released phosphocholine head group, PC-PLC activity uses water as the acceptor (8, 10).

It is well known that D609, a PC-PLC inhibitor, can also inhibit SMS (11, 12). From SMS catalytic activity, we noticed that SMS1- or SMS2-mediated SM formation can be separated into two steps: (1) PC hydrolysis step, where PC is hydrolyzed into P-choline and DAG and (2) SM formation step, where P-choline is added onto ceramide. Besides six membrane-spanning domains, SMS1 and SMS2 also contain four highly conserved sequence motifs, designated D1, D2, D3, and D4 (7). Motifs D3 and D4 contain conserved three amino acids, His-His-Asp, which can form a catalytic triad mediating the nucleophilic attack on the phosphodiester bond of PC (7, 13), that is, the first step of SMS activity. Thus, potentially, SMS1 and SMS2 can be functional PC-PLCs.

In this study, we showed that SMS1 and SMS2 have dual activities, that is, SM biosynthesis and PC-PLC activities, which regulate steady-state levels of PC and DAG as well as SM.

## Results

### SMS1 and SMS2 generate P-choline and DAG *via* the hydrolysis of PC in the absence of ceramide

**Figure 1:**
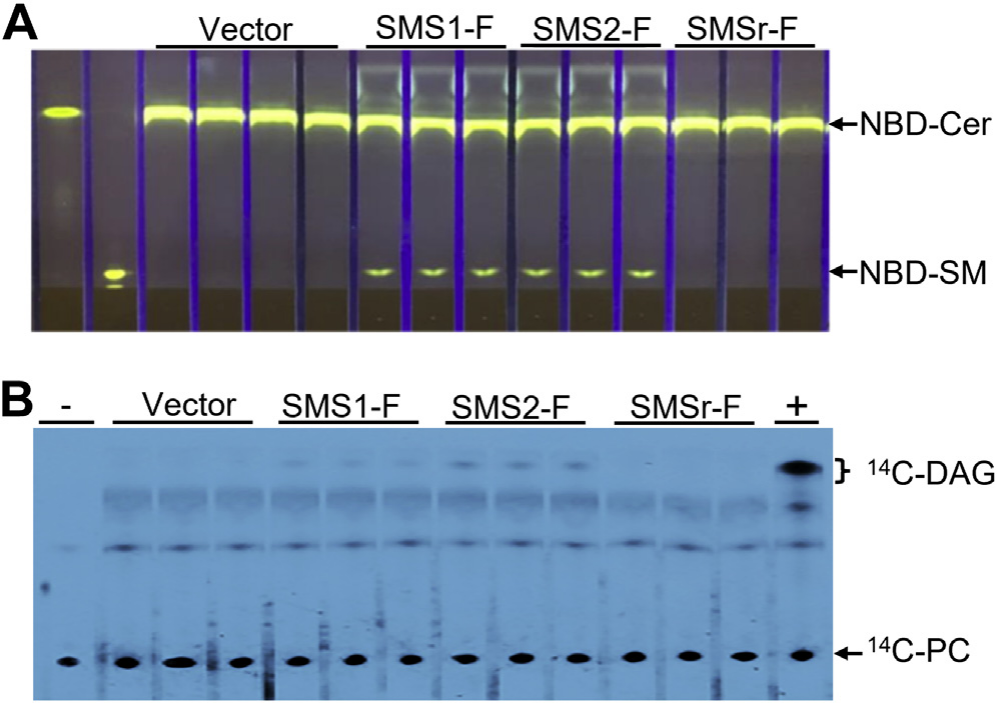
**SMS and PC-PLC activity assays, using SMS immunoprecipitants.** Cos7 cells were transfected with SMS1-flag vector, SMS2-flag vector, SMSr-flag vector, and an empty vector, respectively. SMS-flags (SMS-F)s were immunoprecipitated, and the precipitates were used to perform SM synthase and PC-PLC activity assays, as described in Experimental procedures. The products were separated by TLC. *A*, SM synthase activity assay, using NBD-Cer and PC as two substrates. *B*, PC-PLC activity assay, using ^14^C-PC as a substrate. +, bacterial PLC (20 ng) was used as a positive control; -, blank with no samples; PC, phosphatidylcholine; SMS, sphingomyelin synthase; PC-PLC, PC-phospholipase C.

To examine whether SMS1 and SMS2 have PC-PLC activity, we overexpressed human SMS1-flag, SMS2-flag, and SMSr-flag in Cos7 cells through expression vector transfection, respectively. To avoid the noise from endogenous SMS activity, we immunoprecipitated each flagged SMS using a flag antibody and then utilizing the precipitate to perform SMS activity measurement, using PC and nitrobenz-diazol (NBD)-ceramide as two substrates. We found that both SMS1-flag and SMS2-flag but not SMSr-flag and empty vector had SM synthase activity (Fig. 1*A*).

Next, we utilized the same precipitates to measure PC-PLC activity, using ^14^C-PC, as a substrate (without ceramide). We found that SMS1-flag and SMS2-flag but not SMSr-flag and empty vector produced ^14^C-DAG (Fig. 1*B*). Thus, both SMS1-flag and SMS2-flag have PC-PLC activity, that is, producing DAG through hydrolysis of PC in the absence of ceramide.

**Figure 2:**
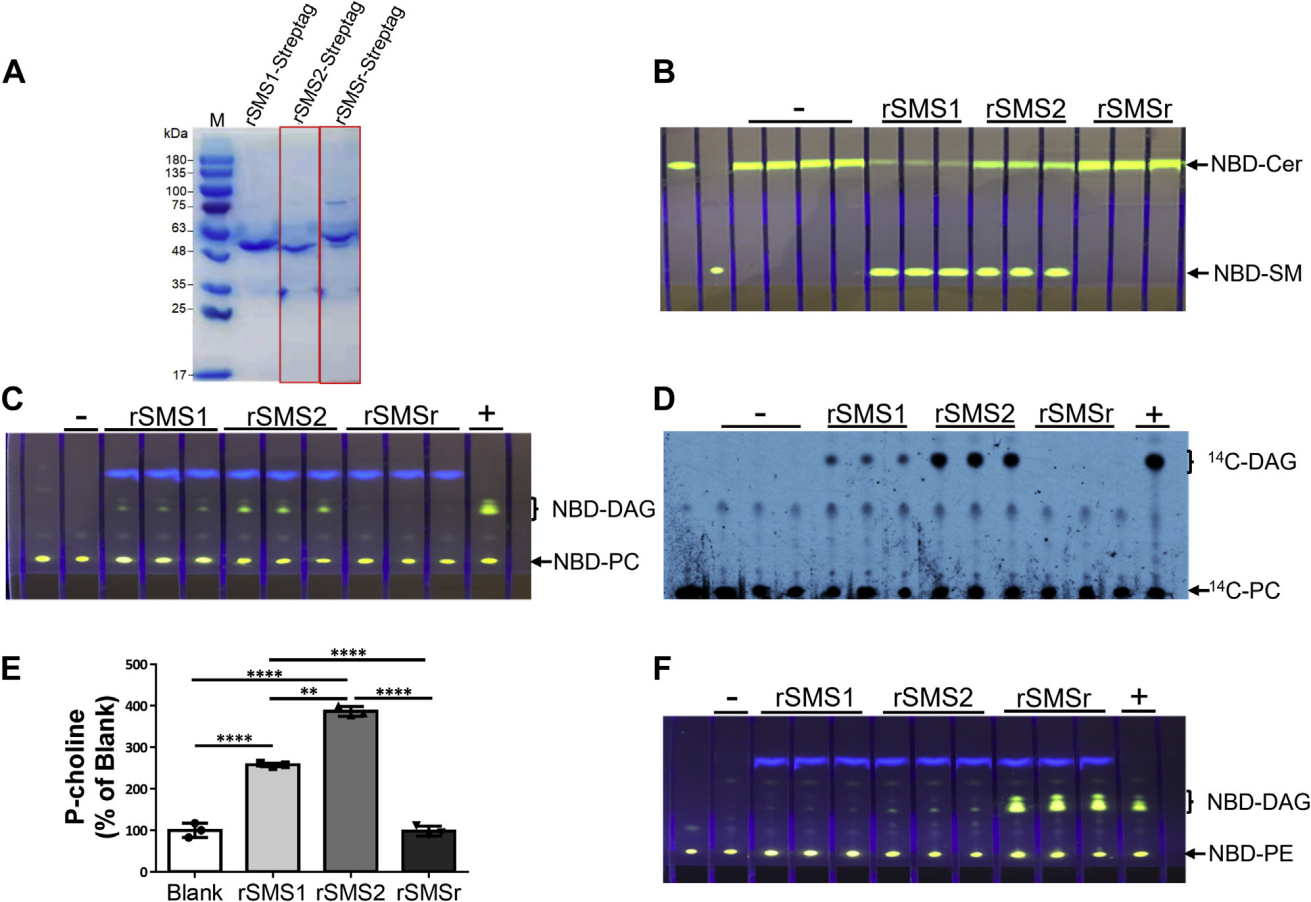
**SMS, PE-PLC, and PC-PLC activity assays, using purified recombinant proteins.** Purified recombinant SMSs (100 ng) were used for enzyme assays. *A*, SDS-PAGE for purified flagged rSMS1, rSMS2, and rSMSr. *B*, SMS activity assay, using NBD-Cer and PC as two substrates. *C*, PC-PLC activity assay, using NBD-PC as a substrate. *D*, PC-PLC activity assay, using ^14^C-PC as a substrate. *E*, direct measurement of P-choline, another product of SMS1- and SMS2-mediated PC-PLC activity. Values represent the mean ± SD, n = 3, ∗∗*p* < 0.01, ∗∗∗∗*p* < 0.0001. *F*, PE-PLC activity assay, using NBD-PE as a substrate. +, bacterial PLC (20 ng) was used as a positive control; -, blank with no samples; PC, phosphatidylcholine; PC-PLC, PC-phospholipase C; PE, phosphatidylethanolamine; SMS, sphingomyelin synthase.

To more precisely show that SMS1 and SMS2 have PC-PLC activity, we expressed recombinant SMS (rSMS) rSMS1–, rSMS2–, and rSMSr–Strep-tag in Baculovirus/*Spodoptera frugiperda* (SF9) cell system and then purified it, using streptavidin affinity column chromatography. As shown in Figure 2*A*, the purity of rSMS1–Strep-tag and rSMS2–Strep-tag was about 96%, while rSMSr–Strep-tag was about 90%. We then utilized the purified rSMS1, rSMS2, and rSMSr to measure SM synthase and PC-PLC activities. We found that rSMS1 and rSMS2 but not rSMSr had SMS activity when NBD-ceramide and PC were used as two substrates (Fig. 2*B*). Importantly, rSMS1 and rSMS2 but not rSMSr had PC-PLC activity when NBD-PC or ^14^C-PC was used, as a substrate, for generating NBD-DAG or ^14^C-DAG (Fig. 2, *C* and *D*). Also, we found that rSMS2 had higher PC-PLC activity than that of rSMS1 when the same amount of recombinant protein was used (Fig. 2, *C* and *D*). Moreover, we directly measured P-choline, another product of PC-PLC, and found that both rSMS1 and rSMS2 produced P-choline, and the latter had a higher activity (Fig. 2*E*).

To evaluate the specificity, next, we sought to measure PE-PLC activity of rSMS1, rSMS2, and rSMSr, using NBD-PE as a substrate. We found that both rSMS1 and rSMS2 had extremely weak or no PE-PLC activity, whereas rSMSr had the high activity (Fig. 2*F*) that was consistent with our previous observation (14).

**Figure 3:**
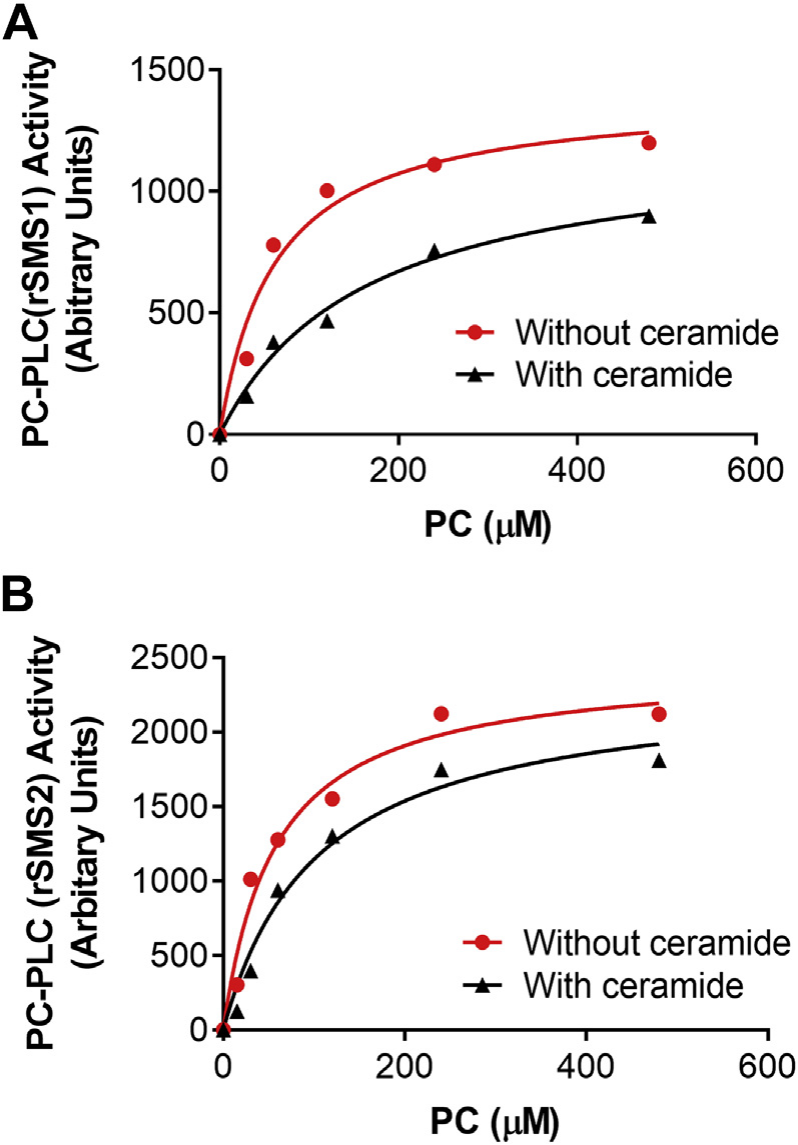
**Michaelis–Menten kinetics of SMS1- and SMS2-mediated PC-PLC reaction.** Purified rSMS1 (50 ng) and rSMS2 (10 ng) were incubated with increased concentration of PC, with/without ceramide (40 μM) at 37 °C for 1 h. Michaelis–Menten constant (*K*_m_) was calculated. *A*, rSMS1-mediated PC-PLC activity. *K*_m_ = 61 μΜ without ceramide and *K*_m_ = 162 μΜ with ceramide. *B*, rSMS2-mediated PC-PLC activity. *K*_m_ = 57 μΜ without ceramide and *K*_m_ = 104 μΜ with ceramide. PC, phosphatidylcholine; PC-PLC, PC-phospholipase C; rSMSs, recombinant SMSs; SMS, sphingomyelin synthase.

We did Michaelis–Menten kinetics of SMS1- and SMS2-mediated PC-PLC reaction. The Michaelis–Menten constant (*K*_m_) was measured. We found that, without ceramide, the *K*_m_ for rSMS1-mediated PC-PLC was 61 μΜ and for rSMS2-mediated PC-PLC was 57 μM. However, both *K*_m_ (162 μM and 104 μM, respectively) became larger when ceramide was added (Fig. 3, *A* and *B*), indicating that both rSMS1- and rSMS2-mediated PC-PLCs have SMS activities.

### Effect of SMS family deficiency on PC and DAG metabolism

**Figure 4:**
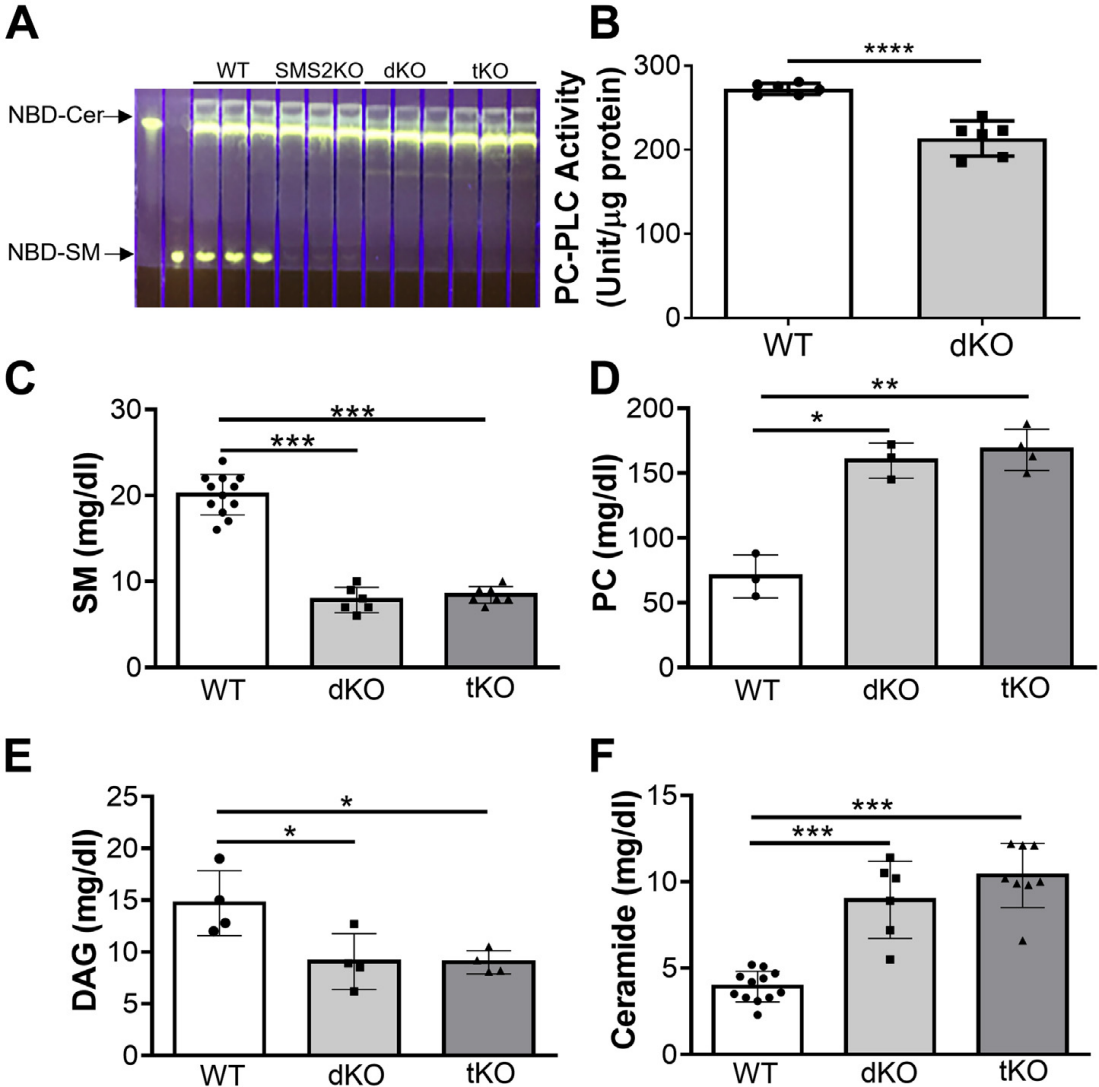
**Mouse plasma lipid measurements.** WT, liver-specific *Sms1*/global *Sms2* double-KO (dKO), and liver-specific *Sms1*/global *Sms2*/global *Smsr* triple-KO (tKO) mice were used. *A*, SMS activity in WT, dKO, and tKO mouse livers. *B*, P-choline measurement–based PC-PLC activity assay. *C*, SM; (*D*) PC; (*E*) DAG; (*F*) ceramide. Values represent the mean ± SD, n ≥ 3, ∗*p* < 0.05, ∗∗*p* < 0.01, ∗∗∗*p* < 0.001, ∗∗∗∗*p* < 0.0001. DAG, diacylglycerol; PC, phosphatidylcholine; PC-PLC, PC-phospholipase C; P-choline, phosphorylcholine; SMS, sphingomyelin synthase.

To prove that SMS1 and SMS2 have PC-PLC activity *in vivo*, we depleted all three members of SMS gene family in mice. We established liver-specific *Sms1*/global *Sms2* double-KO (dKO) mice (15) and crossed them with our global *Smsr* KO mice (16) to obtain liver-specific *Sms1*/global *Sms2*/global *Smsr* triple-KO (tKO) mice. The dKO and the tKO mice have no detectible total SMS activity in their livers (Fig. 4*A*). We also utilized the PC-PLC kit to measure PC-PLC activity in the liver homogenate of WT or dKO mice. We found that the dKO mice had significantly lower liver PC-PLC activity (Fig. 4*B*).

Because the liver is one of the major tissues contributing to plasma lipid metabolism, it is conceivable that the deficiency in SMS/PC-PLC would influence plasma SM, PC, DAG, and ceramide levels. We measured plasma lipids in 2-month-old mice. We found that the dKO and the tKO mice had a significantly lower level of SM (Fig. 4*C*) and a significantly higher level of PC (Fig. 4*D*) than WT mice. However, there were no differences between the dKO and tKO in both lipids (Fig. 4, *C* and *D*), suggesting that SMSr is neither an SMS nor a PC-PLC.

We also noticed that DAG levels were significantly reduced in the dKO and the tKO mice compared with WT mice, whereas no difference between the two KO mouse groups (Fig. 4*E*), suggesting that tissues other than the liver could have effect on DAG level in the circulation. Notably, compared with WT mice, the dKO and the tKO mice had significantly higher ceramide levels (Fig. 4*F*), but there was no significant difference between the dKO and the tKO mice, reflecting that SMSr-mediated CPE synthase activity (using ceramide) *in vitro* could not play an actual role *in vivo*.

**Figure 5:**
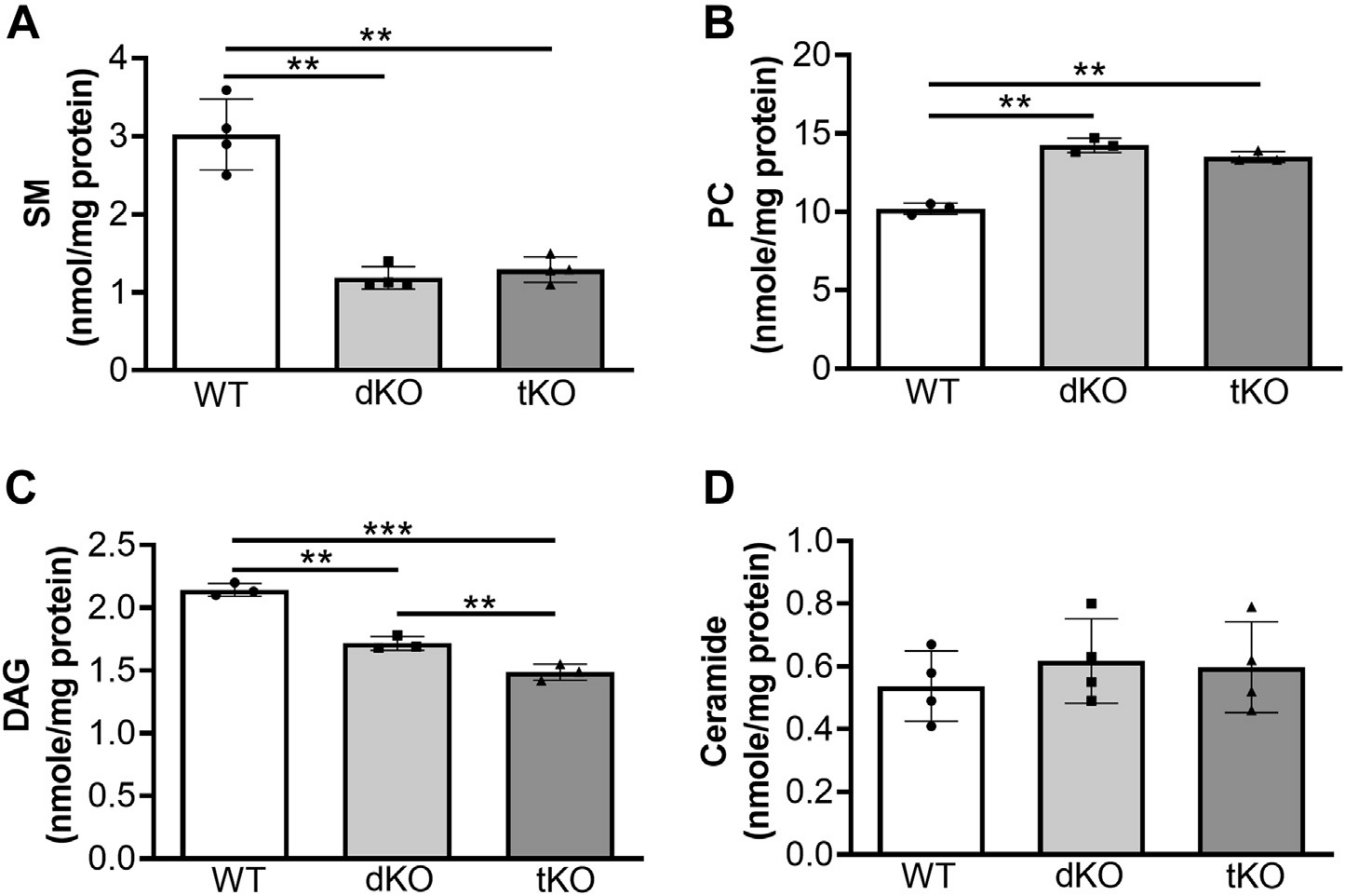
**Mouse liver lipid measurements.** WT, liver-specific *Sms1*/global *Sms2* double-KO (dKO) and liver-specific *Sms1*/global *Sms2*/global *Smsr* triple-KO (tKO) mice were used. *A*, SM; (*B*) PC; (*C*) DAG; and (*D*) ceramide. Values represent the mean ± SD, n = 3 to 4, ∗∗*p* < 0.01, ∗∗∗*p* < 0.001. DAG, diacylglycerol; PC, phosphatidylcholine; SM, sphingomyelin.

We next sought to measure liver lipids. As expected, we found that, compared with WT mice, the dKO mice and the tKO mice had similar reduction of SM (Fig. 5*A*) and a similar induction of PC levels (Fig. 5*B*). Notably, the order of liver DAG levels is WT > dKO > tKO mice, reflecting the reduction of SMS-related PC-PLC and PE-PLC activities (Fig. 2, *C*, *D*, and *F*). Interestingly, although both dKO and tKO mouse livers have more ceramide levels, the difference between WT and dKO or tKO is not statistically significant (Fig. 5*D*).

**Figure 6:**
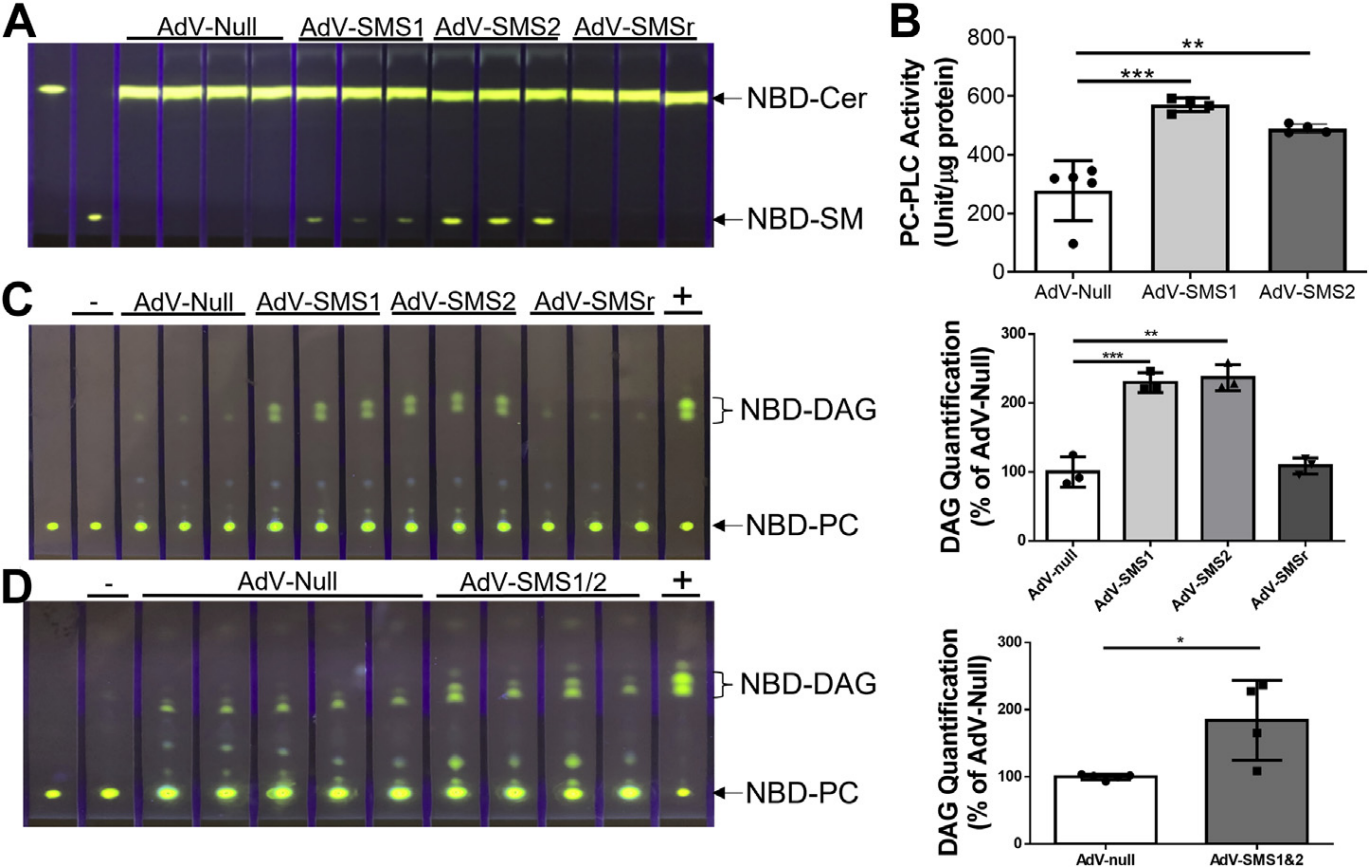
**The effect of AdV-SMSs on *Sms1*/*Sms2*/*Smsr* triple-KO mice**. The tKO mice were injected (i.v.) with AdV-SMS1, AdV-SMS2, AdV-SMSr, and AdV-null (1 × 10^11^ viral particles/mouse), respectively. Four days after the injection, mouse liver homogenates were prepared and used for enzyme assays. *A*, SMS activity assay, using NBD-Cer and PC as two substrates. *B*, PC-PLC activity assay using the PC-PLC kit that measures P-choline formed from PC-PLC reaction. *C* and *D*, PC-PLC activity assay, using NBD-PC as a substrate. +, bacterial PLC (20 ng) was used as a positive control; -, blank with no samples. ∗*p* < 0.05, ∗∗*p* < 0.01, ∗∗∗*p* < 0.001. AdV, adenovirus; PC, phosphatidylcholine; PC-PLC, PC-phospholipase C; SMS, sphingomyelin synthase; SMSr, SMS-related protein; tKO, triple KO.

To further prove that SMS1 and SMS2 have PC-PLC activity *in vivo*, we injected (i.v.) adenovirus (AdV)-SMS1, AdV-SMS2, AdV-SMSr, and AdV-null into 2-month-old tKO mice, respectively. At day 4 after the injection, we isolated the liver from the mice. As expected, AdV-SMS1 and AdV-SMS2 but not AdV-SMSr and AdV-null had SMS activity (NBD-SM as a product) (Fig. 6*A*). We also used the PC-PLC kit to measure PC-PLC activity in the liver homogenate of AdV-null or AdV-SMS1 or AdV-SMS2 mice. We found that the AdV-SMS1 or AdV-SMS2 treatment significantly increased PC-PLC activity in the liver, compared with AdV-null treatment (Fig. 6*B*). Moreover, both AdV-SMS1 and AdV-SMS2 but not AdV-SMSr and AdV-null increased liver PC-PLC activity, when NBD-PC was used as a substrate (Fig. 6*C*). AdV-SMS1/AdV-SMS2 combined injection, again, confirmed the induction of PC-PLC activity (Fig. 6*D*). We also noticed that other enzyme activities can also influence NBD-DAG production because there was a weak NBD-DAG signal in both AdV-SMSr– and AdV-null–treated mouse livers (Fig. 6, *C* and *D*).

**Figure 7:**
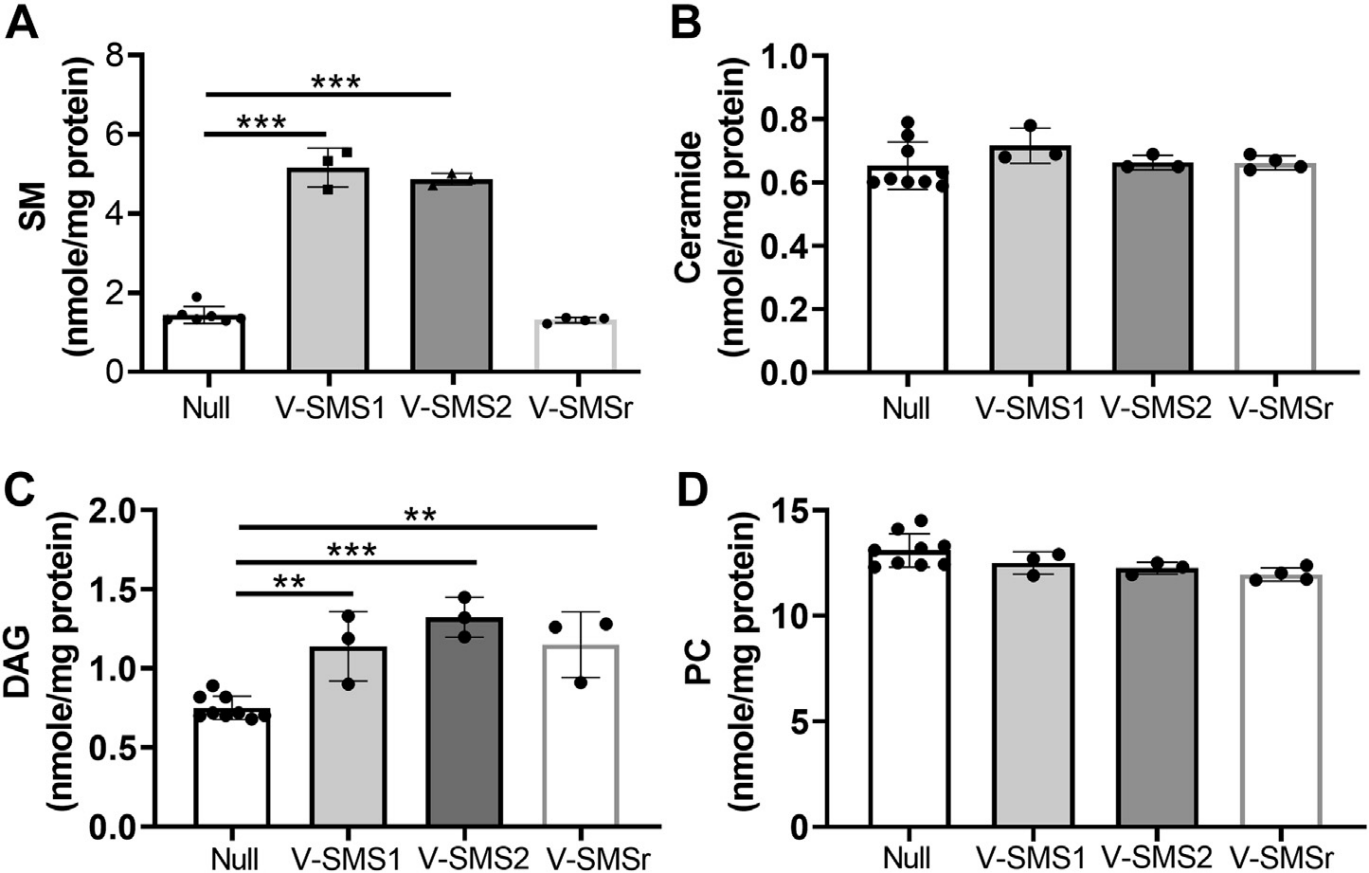
**The effect of AdV-SMSr on *Sms1*/*Sms2*/*Smsr* triple-KO mouse liver lipids.** The tKO mice were injected (i.v.) with AdV-SMSs and AdV-null (1 × 10^11^ viral particles/mouse), respectively. Four days after the injection, mouse liver homogenates were prepared and used for lipid analysis. *A*, SM, (*B*) ceramide, (*C*) DAG, and (*D*) PC. Values represent the mean ± SD, n ≥ 3, ∗∗*p* < 0.01, ∗∗∗*p* < 0.001. AdV, adenovirus; DAG, diacylglycerol; PC, phosphatidylcholine; SMS, sphingomyelin synthase; SM, sphingomyelin; SMSr, SMS-related protein; tKO, triple KO.

Next, we sought to measure liver lipid levels in these AdV-SMS–injected tKO mice. As expected, liver CPE was undetectable in all groups (data not shown). AdV-SMS1 or AdV-SMS2 but not AdV-SMSr treatment dramatically increases SM levels (Fig. 7*A*); however, their effect on ceramide is marginal (Fig. 7*B*). Furthermore, DAG levels were elevated in all AdV-SMS–treated livers (Fig. 7*C*). Although we did not find significant changes of total PC levels (Fig. 7*D*, Table S1), we found that expression of SMS1 or SMS2 in the tKO mice could reduce the high levels of liver PC back to WT levels by comparing Figure 7*D* with Figure 5*B*.

## Discussion

In the present study, we identified that SMS1 and SMS2 exhibit PC-PLC activities. Furthermore, we demonstrated that SMS1/SMS2 deficiency influences steady-state levels of PC and DAG.

One of the key findings of the present study is that SMS1 and SMS2 have PC-PLC activity. PC-PLC is an important member of PLC family to catalyze the hydrolysis of the ester linkage between glycerol and phosphate in PC, producing DAG and P-choline (17). Since the 1990s, a growing body of evidence has pointed to the implication of PC-PLC in metabolism, growth, differentiation, senescence, and apoptosis of mammalian cells (18, 19, 20, 21). Today, a major gap in our knowledge of this important enzyme is linked to the fact that the mammalian PC-PLC has not been cloned, although bacterial PC-PLC was cloned (5). Thus, so far, PC-PLC studies in mammalian cells and *in vivo* have to rely on small-molecule inhibitors of PC-PLC, such D609 (6) and so on (17). A question was asked more than 20 years ago: does SMS account for the putative PC-PLC? (12). Given the fact that D609 can inhibit both PC-PLC and SMS activities (12), we hypothesize that SMS1 and SMS2 have both PC-PLC and SMS activities. Indeed, so far, we have clear-cut evidence to show that both SMS1 and SMS2 have PC-PLC activity.

By measuring DAG, as one of the products, we found that in a tissue culture system, immunoprecipitated flag-tagged SMS1 and SMS2 had PC-PLC activity (Fig. 1*B*). Importantly, we found that purified rSMS1 and rSMS2 had PC-PLC but not PE-PLC activity (Fig. 2, *C*, *D*, and *F*). Moreover, we used AdV to express SMS1 and SMS2 in the liver of *Sms1/Sms2/Smsr* tKO mice and found that AdV-SMS1 and AdV-SMS2 treatment induced liver PC-PLC activity (Fig. 6, *C* and *D*). A weak NBD-DAG signal in both AdV-SMSr– and AdV-null–treated mouse livers was probably caused by enzymes other than SMS family (Fig. 6, *C* and *D*).

By measuring P-choline, as another product, we found that rSMS1 and rSMS2 had PC-PLC activity (Fig. 2*E*) with comparable *K*_m_ (Fig. 3, *A* and *B*). The *K*_m_ became larger when ceramide was present. Furthermore, we found that PC-PLC activity was significantly reduced in the dKO liver (Fig. 4*B*), compared with the control. In addition, we noticed that the dKO mouse liver still contained substantial levels of P-choline, suggesting that enzymes other than SMS1 and SMS2 could be responsible for its formation. Furthermore, AdV-SMS1 or AdV-SMS2 treatment significantly induced PC-PLC activity in the tKO mouse liver (Fig. 6*B*).

Another key finding of the present study is that SMS1 and SMS2 are involved in regulating steady-state levels of DAG and PC. We measured SM, PC, DAG, and ceramide in WT, *Sms1*/*Sms2* dKO, and *Sms1*/*Sms2*/*Smsr* tKO mouse plasma and liver. The dKO and the tKO mice have a similar reduction of SM levels in the plasma and liver, indicating that SMS1 and SMS2 but not SMSr are SMS (Figs. 4*C* and 5*A*) (7, 22). Furthermore, the dKO and the tKO mice have a similar accumulation of PC levels, compared with WT mice, in the plasma and liver, demonstrating that SMS1 and SMS2 but not SMSr have PC-PLC activity (Figs. 4*D* and 5*B*).

In addition, we found that plasma DAG levels are significantly reduced in the dKO mice (Fig. 4*E*), compared with WT, and the difference between the dKO and the tKO mice is marginal. The reason could be related with many DAG-associated metabolisms (23). DAG levels in the liver were proportionally reduced in the order of WT, dKO, and tKO (Fig. 5*C*), indicating that all SMS family members contribute to DAG levels in the liver because of their PC-PLC and PE-PLC activities (Fig. 2, *C*, *D*, and *F*). Although DAG is known as an activator of certain PKCs (24), DAG generated from different sources may have variable effects on PKCs. For example, DAG derived from hydrolysis of phosphatidylinositol 4,5 biphosphate can activate PKCs, whereas DAG produced from other sources cannot activate PKCs (25). The impact of PC-PLC–related DAG deserves further investigation.

Furthermore, we found that the dKO mice have significantly higher ceramide levels than WT mice in the plasma (Fig. 4*F*), reflecting that both SMS1 and SMS2 deficiency prevents using ceramide as a substrate to produce SM (7, 22). However, SMSr deficiency has no impact on plasma ceramide levels, indicating SMSr is not a functional CPE synthase (*in vivo*) that consumes ceramide (9).

Injection of AdV-SMSs into the tKO mice allowed us to see the effect of SMS-mediated PLCs from a new perspective. No detectable CPE in the tKO mouse livers (data not shown) was observed after AdV-SMS injection. This result indicated that SMSs are not functional CPE synthase *in vivo*, although we found that all SMSs have this activity when NBD-ceramide and PE (as two substrates) were used in test tubes (16). DAG induction in the liver was observed in all three AdV treatments, which also indicated that all SMS family members play roles in controlling liver DAG levels (Fig. 7*C*).

Very recently, Murakami and Sakane have shown that SMSr can generate DAG *via* the hydrolysis of glycerophospholipids, including PE, PC, and phosphatidic acid, in the absence of ceramide (26). Our present study together with Murakami and Sakane's study (26), as well as our recent report (14), clearly indicated that all three SMS family members are a group of PLCs. Our studies further indicated that their PLC activities have specificities. SMS1 and SMS2 have PC-PLC activity, consisting with their SMS activity (7, 8), whereas SMSr has PE-PLC activity (14), consistent with its *in vitro* CPE synthase activity (9).

Although the changes in PC and DAG levels observed in *Sms* KO mice are in line with PC-PLC activity of SMS1 and SMS2 detected by enzymatic assays *in vitro*, further work will be necessary to determine to what extent these changes are truly mediated by their PC-PLC activity *in vivo*.

We conclude that SMS1 and SMS2 can generate DAG and P-choline through the hydrolysis of PC in the absence of ceramide. Our data indicate that SMS1 and SMS2 are two candidates for the long-sought PC-PLC(s) that can be inhibited by D609 (11, 12). The biological and pathological functions of SMS-mediated PC-PLC activity deserve further investigation.

## Experimental procedures

### Mice

Because it is not possible to obtain global *Sms1/Sms2/Smsr* tKO mice by simply crossing all three single global KO mice together, we crossed liver-specific *Sms1*/global *Sms2* dKO mice (15) with global *Smsr* KO mice (16), yielding liver-specific *Sms1* KO/global *Sms2*/global *Smsr* tKO mice. All animals are bred on a C57BL/6 genetic background. Experiments involving animals were conducted with the approval of the State University of New York Downstate Medical Center Institutional Animal Care and Use Committee. The procedures followed were in accordance with institutional guidelines.

### Tissue culture and transfection

Cos7 cells were maintained in the growth medium (Dulbecco's modified Eagle's medium with 10% fetal bovine serum, 2 mM L-glutamine, 1 mM sodium pyruvate, 100 units/ml penicillin, and 100 μg/ml streptomycin) at 37 °C in 5% CO_2_. Cos7 cells overexpressing SMSs were established by transfection using Lipofectamine 2000 (Invitrogen) with p3xflags-cmv-13 vectors (Sigma) containing SMSr. All cells were cultured at 37 °C in 5% CO_2_. The harvested cells were stored at −80 °C until use.

### SMS-flag immunoprecipitate preparation

The SMS1-flag or SMS2-flag or SMSr-flag–transfected cells were homogenized in the buffer (5% sucrose, 50 mM Tris HCl, and 1 mM EDTA) containing a protease inhibitor cocktail (Roche Diagnostics). Supernatants were transferred to new tubes after centrifugation at 1000*g* for 10 min at 4 °C and stored at −80 °C until use. Protein concentrations of the homogenates were determined with a BCA Protein assay (Pierce) using BSA as the standard. Different from liver homogenates, the supernatants from cells (∼1000 μg protein) were further incubated with 20 μl of resuspended volume of protein A/G plus-agarose (Santa Cruz) at 4 °C for 30 min. After spinning at 8200*g* for 30 s at 4 °C, the supernatants were then incubated with 30 μl of resuspended volume of EZView red anti-flag M2 affinity gel for overnight at 4 °C. Finally, extracted SMSs were isolated by centrifugation at 8200*g* for 30 s at 4 °C for enzyme activity assays.

### rSMS preparation

The cDNA for human SMS1, SMS2, and SMSr was, respectively, cloned into an expression vector modified from pFastBac Dual, with a Strep-tag II amino acid sequence (Trp-Ser-His-Pro-Gln-Phe-Glu-Lys) and then a tobacco etch virus protease recognition sequence added to the N terminus of cDNA of SMSs. The SMS proteins were expressed using a Baculovirus/*S. frugiperda* (SF9) cell system. The rSMSs were purified according to a published protocol (27). In principle, the Strep-tag was developed as an affinity tool for the purification of fusion SMSs on streptavidin affinity column. After purification, the Strep-tag could be removed by tobacco etch virus protease hydrolysis for other studies. However, we used the tagged SMSs for both SMS and PLC activity assays.

#### SMS and CPE assays

The procedure was as reported (16). Blank indicates the reaction without SMS-flag and rSMS.

#### PC-PLC and PE-PLC assays

In principle, P-choline or P-ethanolamine has two acceptors, that is, ceramide and water. If the acceptor is ceramide, the reaction represents SMS activity. If the acceptor is water, the reaction represents PLCs. SMS-flag purified by immunoprecipitation or rSMS was incubated with the reaction buffer (50 mM Tris HCl, pH 7.4, 140 mM NaCl, 10 mM dimethyl glutarate, 2 mM CaCl_2_) and 2 μg NBD-PC/NBD-PE (16:0–06:0, Avanti Polar Lipids) in total volume of 500 μl as well as replacing NBD substrates with 0.25 μCi [^14^C]-PC (American Radiolabeled Chemical) for incubation. Blank indicates the reaction without SMS-flag and rSMS. The reactions were incubated at 37 °C water bath for 1 h and then stopped by adding 500 μl of chloroform-methanol (2:1 v/v) with vigorous vortex. The lipids were then extracted and dried. For analysis, the dried lipid phase was redissolved in a 20 μl of chloroform and applied to a TLC plate (Silica gel, Whatman). NBD or [^14^C] substrates and generated NBD- or [^14^C]-DAG were separated using a basic eluent (chloroform-methanol, 15:1, v/v). After thoroughly drying the TLC plate, the NBD-lipid species were visualized under the UV light, whereas ^14^C-lipid species were visualized after exposure on film, which were contrast-adjusted using the Photoshop software after scanning.

#### P-choline measurement–based PC-PLC activity assay

PC (8 μg) was incubated with rSMS1, rSMS2, or rSMSr (150 ng) or mouse liver homogenate (20 μg protein) in 200-μl working solution (200 μM Amplex Red reagent containing 2 U/ml HRP, 8 U/ml alkaline phosphatase, 0.2 U/ml choline oxidase) at 37 °C water bath for 1 h. For *K*_m_ measurement, gradient-increased PC (0, 15, 30, 60, 120, 240, 480 μM) were incubated rSMS1 or rSMS2 (150 ng), with or without 2 μg ceramide, in the working solution at 37 °C water bath for 1 h. The generated P-choline was measured by the Amplex Red PC-PLC–specific assay kit (Molecular Probes Inc), modified as described (28).

### Lipid measurements

PC, DAG, and SM were measured by a PC Assay Kit (BioVision, Cat# K576), a DAG Assay Kit (Cell Biolabs, Cat# MET-5028), and an SM Assay Kit (BioVision, Cat# K600), following the corresponding protocols, respectively. Ceramide was measured by LC/MS/MS at the University of Taxes San Antonio, on the fee-for-service basis.

### AdV administration

AdV-SMSs were prepared by ViraQuest Inc. *Sms1/Sms2/Smsr* tKO mice were injected (i.v.) with AdV-SMSs (1 × 10^11^ viral particles/mouse), respectively. AdV-null was used as the control. At day 4 after the injection, mouse livers and plasma were collected for enzyme and lipid analyses.

### Statistical analysis

Each experiment was conducted at least two times. Data were expressed as the mean ± SD. Differences between two groups were analyzed by the unpaired, two-tailed Student's *t* test, and differences among multiple groups were assessed by ANOVA, followed by the Student-Newman-Keuls test. *p* values < 0.05 were considered significant.

## Data availability

All data are contained within the article.

## Supporting information

This article contains supporting information.

## Conflict of interest

The authors declare that they have no conflicts of interest with the contents of this article.
