# Supplementary material for: Sphingomyelin synthases 1 and 2 exhibit phosphatidylcholine phospholipase C activity
Source: J Biol Chem. 2021 Nov 10;297(6):101398. doi: 10.1016/j.jbc.2021.101398 (PMC8648842; doi:10.1016/j.jbc.2021.101398)
Supplement: Table S1 [file mmc1.pdf]

Supplement Table 1

Measurement of PC subspecies in tKO liver with AdV-SMSs treatment

PC subspecies

nmol/mg protein	D16:1-16:1	D16:1-16:0	D16:0-16:0	P16:0-18:1	P16:0-18:0	D16:1-18:2	D16:0-18:2	D16:0-18:1	D16:0-18:0	P18:1-18:1	P18:0-18:1	A18:0-18:1	D18:2-18:3	D18:2-18:2	D18:1-18:2	D18:0-18:2	D18:0-18:1
AdV-Null	0.03±0.01	0.25±0.04	0.59±0.04	0.04±0.01	0.04±0.03	0.78±0.06	10.86±2.26	3.93±0.33	0.06±0.01	0.04±0.03	0.28±0.04	2.89±0.59	0.99±0.05	8.01±1.26	3.53±0.61	5.8±1.25	0.47±0.06
AdV-SMS1	0.06±0.01	0.33±0.02	0.54±0.07	0.04±0.03	0.08±0.02	1.03±0.12	9.49±0.34	4.96±0.53	0.07±0.01	0.07±0.02	0.35±0.07	2.76±0.5	0.76±0.18	5.96±0.95	3.56±0.57	5.17±0.37	0.66±0.07
AdV-Null	0.04±0.01	0.19±0.05	0.71±0.15	0.05±0.02	0.06±0.02	0.54±0.06	9.09±1.61	3.41±0.02	0.07±0.04	0.04±0.01	0.27±0.06	2.99±1.55	0.86±0.13	6.29±0.65	2.94±0.09	5.94±0.88	0.51±0.1
AdV-SMS2	0.05±0.02	0.25±0.05	0.59±0.05	0.02±0.03	0.06±0.04	0.63±0.12	8.08±1.41	3.75±0.35	0.09±0.02	0.04±0.02	0.3±0.09	2.65±0.53	0.59±0.03	5.62±0.63	2.47±0.38	5.44±0.42	0.61±0.07
AdV-Null	0.05±0.01	0.23±0.03	0.33±0.07	0.07±0.02	0.11±0.01	0.7±0.05	9.86±0.95	3.48±0.33	0.07±0.01	0.06±0.02	0.33±0.05	2.14±0.43	1.05±0.06	6.81±0.49	2.26±0.22	4.22±0.7	0.38±0.05
AdV-SMSr	0.02±0.004	0.21±0.01	0.32±0.03	0.06±0.03	0.13±0.07	0.59±0.04	9.41±0.63	3.7±0.24	0.04±0.01	0.06±0.03	0.31±0.04	1.87±0.16	0.65±0.12	6.6±0.58	1.96±0.14	3.52±0.12	0.44±0.05

PC subspecies

nmol/mg protein	P18:1-20:4	P18:0-20:4	P18:2-20:1	P18:1-20:1	P18:0-20:1	D18:2-20:5	D16:0-22:6	D18:1-20:4	D18:2-20:2	D18:0-20:3	D18:0-20:2	A18:0-22:6	D18:2-22:6	D18:1-22:6	D18:0-22:6	D18:0-22:5
AdV-Null	0.07±0.01	0.1±0.01	0.34±0.06	2.28±0.32	0.72±0.22	0.39±0.07	6.75±0.84	2.24±0.59	4.71±0.51	0.77±0.07	0.09±0.04	0.12±0.04	0.14±0.01	1.33±0.19	2.21±0.4	0.19±0.04
AdV-SMS1	0.06±0.01	0.1±0.01	0.27±0.08	1.94±0.52	0.75±0.21	0.42±0.08	4.7±0.31	2.1±0.34	3.78±0.37	0.68±0.12	0.11±0.05	0.13±0.02	0.17±0.03	1.26±0.18	2.07±0.08	0.18±0.04
AdV-Null	0.11±0.04	0.18±0.02	0.37±0.05	1.95±0.38	0.63±0.15	0.34±0.06	5.97±0.59	2.21±0.21	5.35±0.99	0.58±0.06	0.11±0.05	0.14±0.03	0.14±0.01	1.42±0.05	2.77±0.25	0.18±0.09
AdV-SMS2	0.05±0.01	0.14±0.05	0.26±0.04	1.96±0.29	0.65±0.15	0.33±0.06	5.78±0.51	1.78±0.14	5.4±0.6	0.65±0.03	0.12±0.02	0.14±0.03	0.14±0.01	1.16±0.06	3.08±0.25	0.16±0.02
AdV-Null	0.17±0.02	0.2±0.03	0.38±0.07	1.61±0.4	0.47±0.12	0.3±0.02	6.48±0.57	1.52±0.09	3.25±0.26	0.51±0.08	0.11±0.02	0.08±0.05	0.24±0.02	0.72±0.06	1.55±0.09	0.12±0.02
AdV-SMSr	0.13±0.02	0.2±0.05	0.23±0.04	1.31±0.22	0.42±0.1	0.22±0.03	5.43±0.39	1.22±0.16	2.84±0.35	0.38±0.04	0.08±0.02	0.14±0.06	0.23±0.03	0.65±0.05	1.53±0.1	0.1±0.01
